# Focus movement distance per pulse dependence of electrical conductivity and diameter of diamond internal modification induced by picosecond laser

**DOI:** 10.1038/s41598-022-21432-9

**Published:** 2022-10-31

**Authors:** Daijiro Tokunaga, Masataka Sato, Sho Itoh, Hirofumi Hidai, Takashige Omatsu, Souta Matsusaka

**Affiliations:** 1 Department of Mechanical Engineering, Chiba, Japan; 2 Molecular Chirality Research Center, Chiba University, Chiba, Japan

**Keywords:** Engineering, Optics and photonics

## Abstract

Internal and local modifications via ultrashort pulsed laser illumination to diamond are promising for manufacturing diamond electronic devices. The relationship between the diameter/electrical conductivity of modified regions and the laser fluence distribution was investigated. Picosecond laser illumination without scanning the laser focus fabricated short modified regions in diamond. As a result, the calculated laser fluence distribution matches the distribution of the modified regions. Wire-shaped modified regions were fabricated via laser illumination with scanning of the laser focus, and the corresponding diameter and electrical conductivity were investigated by controlling the laser focus movement distance per pulse (*V*_*f*_). The modified regions fabricated with varying *V*_*f*_ were divided into three categories depending on the trend of the relationship between the diameter and electrical conductivity. The diameters of the modified regions were constant at the maximum values when *V*_*f*_ was sufficiently small, decreased with increasing *V*_*f*_, and reached a minimum when *V*_*f*_ was sufficiently large. The modified regions became more electrically conductive with increasing *V*_*f*_, even when the deposited energy per unit length decreased. Moreover, the electrical conductivity decreased significantly when the diameter became constant at the minimum value. Finally, the relationship between the diameter/electrical conductivity of the modified regions and the laser fluence distribution was elucidated.

## Introduction

Diamonds possess superior properties for use in many applications, such as abrasive grains and cutting tools (utilizing the hardness of diamonds)^1^, as well as in quantum information devices as sensors for electron spin (utilizing the nitrogen-vacancy center created inside a diamond)^2^. Owing to their high thermal conductivities and dielectric strengths, diamonds are expected to be used as semiconductors in high-power devices. Many studies have been conducted on diamond semiconductors, including the development of a large-scale diamond synthesis method based on chemical vapor deposition^3–5^.

It has been reported that use of a focused ultrashort-pulsed laser can locally modify the inside of a diamond via multiphoton absorption^6,7^. This modified region grows toward the laser source, and a wire-shaped modified region is then formed via laser-focus scanning^8,9^. The modified region constitutes amorphous carbon (a-C) and is electrically conductive^10,11^. This graphitization inside the diamonds was confirmed through observations of the cross section of the modified wire-shaped region via Raman spectroscopy and electron energy loss spectroscopy^12–14^. Picosecond (ps) laser illumination inside a diamond efficiently graphitizes diamond compared with femtosecond laser illumination^15^. The shape of the modified regions can be controlled to form three-dimensional structures by varying the laser focus-scanning direction^16,17^. The internally modified regions of diamonds are expected to be used in high-power electrical device, photonic crystal, and photodiode applications, among others.

Kononenko and Ashikkalieva developed a modification mechanism model for inside a diamond by measuring the growth rate of the modified region^7,18^. Moreover, the shapes, such as the diameter and length, and electrical conductivity of the modified regions were controlled by varying the laser parameters^10,11^. However, the detailed relationship between the diameter/electrical conductivity of the modified regions and the laser parameters has not been studied. Fabricating high-aspect-ratio and electrically conductive modified regions is essential for manufacturing small integrated applications suitable for the development of electrical devices. In addition, fabricating the modified region using high-speed laser scanning is preferable for efficient manufacturing of electrical devices.

In this study, a wire-shaped modified region fabricated at a higher laser scanning speed was found to be electrically conductive. Interestingly, this result indicates that a smaller deposited energy per length converts diamond to graphite more efficiently. The diameter and electrical conductivity of the wire-shaped modified regions fabricated via picosecond laser scanning were investigated. In addition, the diameters of the wire-shaped modified and short-modified regions fabricated without laser focus scanning were compared.

## Results and discussion

### Shape of short modified region

**Figure 1:**
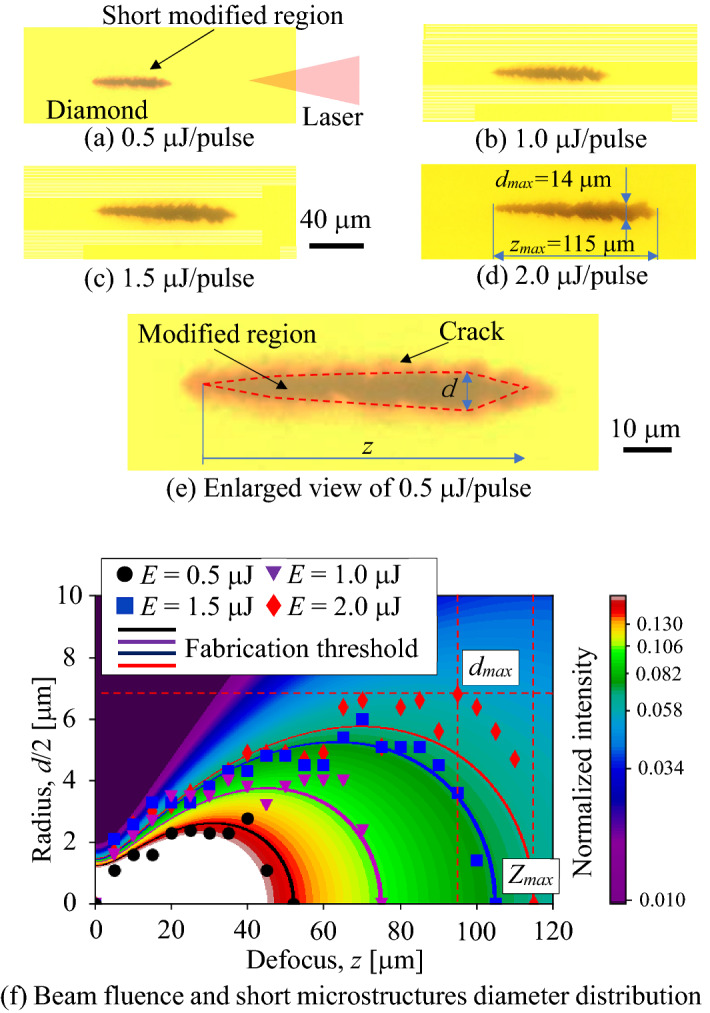
Micrographs of short modified regions and relationship between diameter of short modified regions and beam fluence distribution.

Figure 1a–d show micrographs of the short modified regions fabricated with laser energies of 0.5–2.0 μJ without scanning the laser focus. The short modified regions were spindle-shaped regardless of the laser pulse energy. The modified region lengthened along the beam axis as laser pulse energy increased. Figure 1e shows an enlarged view of Fig. 1a. The modified region consists of a black region in the center of the modified region, indicated by the broken red line, and a light-colored region around the black region. The black region represents a modified region consisting of a-C. In contrast, the light-colored region is a crack region formed by the internal stress generated by volume expansion during modification. In this study, the diameter *d* of the modified region was defined using the diameter of the black region and not the light-colored region shown in Fig. 1e.

The modification start point inside the diamond corresponds to the focal point, regardless of the laser energy, because multiphoton absorption is essential for initiating the modification. The modified region and the laser fluence distribution were compared by assuming that the modification start point was the focal point. The laser fluence distribution was calculated under the following conditions: the Gaussian distribution of the laser fluence was calculated using an M^2^ factor of 1.5, numerical aperture (NA) of 0.4, and refractive index of the diamond sample of 2.42. The effects of the aberrations were ignored. The fluence distribution was normalized and the maximum intensity at the focal point was set to 1. Figure 1f shows the radii of the modified regions and half of the axisymmetric laser-fluence distributions. The color bar indicates normalized intensity. The plots show the radius of each modified region, as presented in Fig. 1a–d. The horizontal axis “Defocus, *z*” in Fig. 1f represents the distance from the tip of the modified region, as in Fig. 1e. The colored lines indicate the contour lines of the fabrication threshold for each laser pulse energy calculated using Eq. (1), as described below. The shapes of the modified regions are highly reproducible. For example, the diameter and length errors of the short modified region fabricated with the pulse energy of 2.0 μJ were within ± 0.8 and ± 1.5 μm from the average, respectively. These values are sufficiently small, and the error bars for the shape of the modified regions were omitted. The shape of the modified regions matches the contour lines, regardless of the pulse energy. The experimental maximum diameter *d*_*max*_ and length *z*_*max*_ of the modified region were measured as 14 and 115 μm, respectively.

The laser fluence threshold used to grow the modified region corresponded to the fluence at the laser source-side tip of the modified region on the laser light axis. Thresholds were compared for pulse energies of 0.5–2.0 μJ. Equation (1) determines the fluence *F* applied to a circle with radius *r* centered on the optical axis at defocus position *z* when the radius of the Gaussian beam is defined as *w*(*z*).

1 $$F = \frac{E}{{\pi r^{2} }}\left[ {1 - exp\left( {\frac{{ - 2r^{2} }}{{w\left( z \right)^{2} }}} \right)} \right]$$

**Figure 2:**
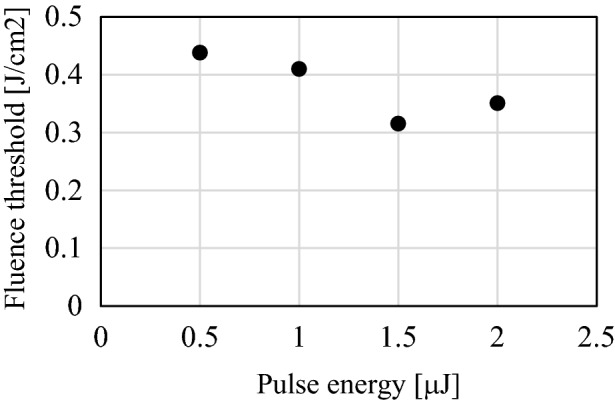
The relationship between pulse energy and fluence threshold.

The limit of *r* = 0 at the tip of the modification indicates the fluence threshold for the modified region. Figure 2 shows the fabrication fluence thresholds for each pulse energy. This figure indicates that the fluence threshold is almost constant regardless of the laser pulse energy, with the average of approximately 0.37 J/cm^2^. This value matches well with that reported in a previous study^7^.

### Shape of wire-shaped modified region

#### Effect of laser repetition rate

**Figure 3:**
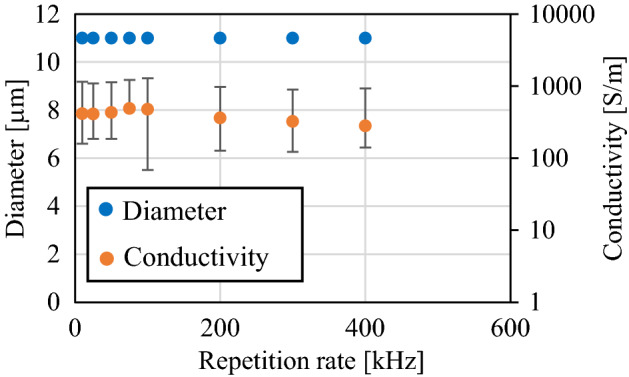
The relationship between pulse repetition rate and diameter and conductivity of wire-shaped modified region under several focus scanning rate.

To reveal the effects of heat accumulation by high-repetition laser illumination (~ 400 kHz)^19^, wire-shaped modified regions were formed by changing the focus scanning speed and laser repetition rate of 10–400 kHz simultaneously, while the laser focus movement distance per pulse *V*_*f*_ in diamond was maintained at 0.48 μm/pulse. The value of *V*_*f*_ was defined as the stage scanning speed (μm/s) divided by the laser repetition rate (Hz) and multiplied by the diamond refraction index of 2.42. The experiment was repeated three times under each condition. Figure 3 displays the diameter and electrical conductivity results of the modified region with varying laser repetition rate. The conductivity plots show the average values of the three samples. The diameter and average electrical conductivity of the wire-shaped modified region were almost constant regardless of the laser repetition rate. Hence, it was concluded that no heat accumulation affected the modification in this range of laser repetition rates. More specifically, the diameter and electrical conductivity of the modified regions would be the same under the same *V*_*f*_ value, even for different combinations of scanning speeds and repetition rates. Therefore, the value of *V*_*f*_ was used as the experimental parameter of the wire-shaped modified region, even with different combinations of repetition rates (10–400 kHz) and scanning speeds.

#### Wire-shaped modified region with various ***V***_***f***_

**Figure 4:**
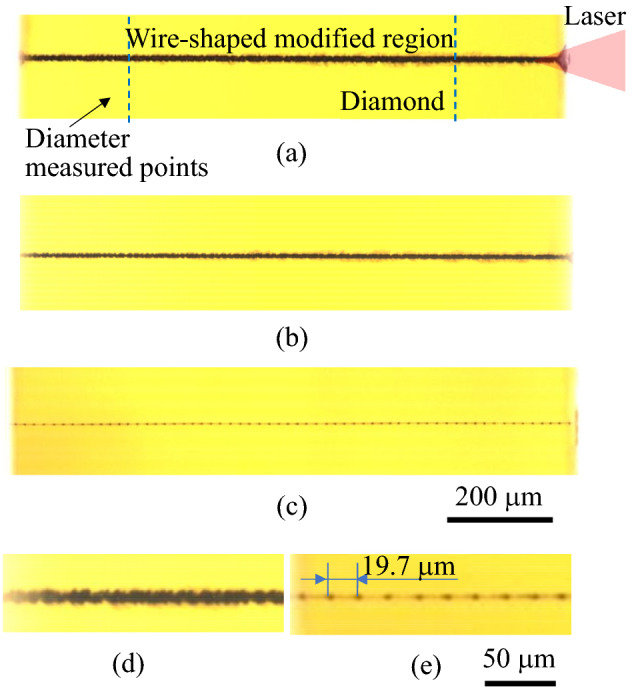
Micrographs of typical wire-shaped modified regions fabricated by different *V*_*f*_: (**a**) 0.16 μm/pulse, (**b**) 4.84 μm/pulse and (**c**) 19.36 μm/pulse. (**d**) and (**e**) are enlarged view of (**b**) and (**c**), respectively.

Figure 4 presents micrographs of the typical wire-shaped modified region formed by *V*_*f*_ = (a) 0.16, (b) 4.84, and (c) 19.36 μm/pulse. The laser pulse energy was set to 2.0 μJ. Figure 4d,e show enlarged views of Fig. 4b,c, respectively. Because the diameters of the modified region at the laser-illuminated surface and the opposite side surface were different, the diameter was calculated using the average values measured at 20% and 80% from the laser-illuminated surface of the total length of the wire-shaped modified region, as indicated by the broken lines in Fig. 4a. The diameter of the modified region decreases with an increasing *V*_*f*_. Laser illumination constantly fabricated the dotted modified region, as shown in Fig. 4e. The interval between the dotted modified regions is approximately 19.67 μm, corresponding to the value of *V*_*f*_ = 19.36 μm/pulse.

**Figure 5:**
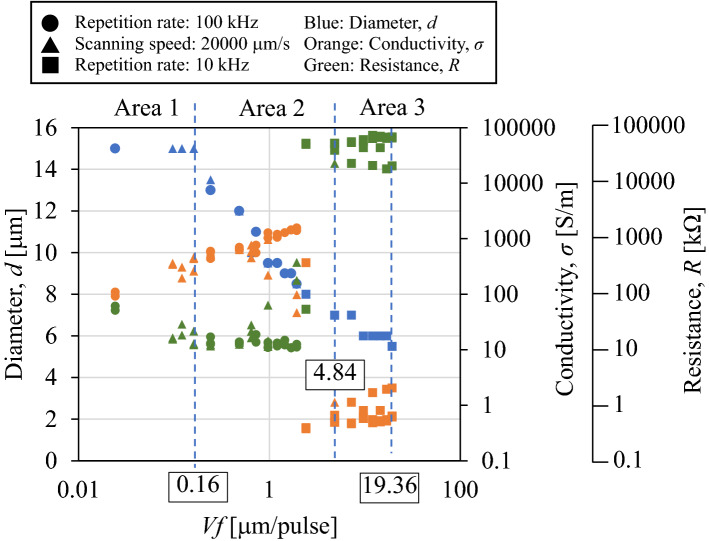
Relationship between *Vf* and the diameter/conductivity/resistance of wire-shaped modified regions.

Figure 5 shows the influence of *V*_*f*_ on diameter *d*, electrical conductivity *σ*, and resistance *R* of the wire-shaped modified region. The shapes of the legends indicate the constant parameter, repetition rate, and scanning speed when *V*_*f*_ was varied. Discussing the effect of the laser parameters on the resistance is challenging because the resistance depends on the diameter. Hence, the electrical conductivity was calculated using the diameter and resistance, and the relation between the conductivity and diameter was employed for the following discussion. The graph can be categorized into three areas (Areas 1–3, shown on the upper side of Fig. 5), depending on the relationship between the diameter and conductivity of the modified regions.

The diameter was constant at a maximum value of approximately 15 μm in Area 1. The diameter corresponded to the maximum diameter of the short modified region fabricated under the same laser energy (in the case where the focus was not scanned). The diameter monotonically decreased with increasing *V*_*f*_ in Area 2, and remained constant at the minimum value of 6 μm in Area 3. This result shows that the laser fluence distribution determines the shape of the wire-shaped modified region in the same manner as that of the short modified region.

Figure 5 shows that the electrical conductivity increases with the value of *V*_*f*_ in Areas 1 and 2 and markedly decreases in Area 3. The electrical conductivity of the wire-shaped modified region increases with longer *V*_*f*_, despite the almost constant diameter in Area 1. The modified regions comprise the sp^2^ and sp^3^ bonds (a-C). Higher laser fluence illumination converts more sp^2^ bonds from sp^3^-bonded diamond.

**Figure 6:**
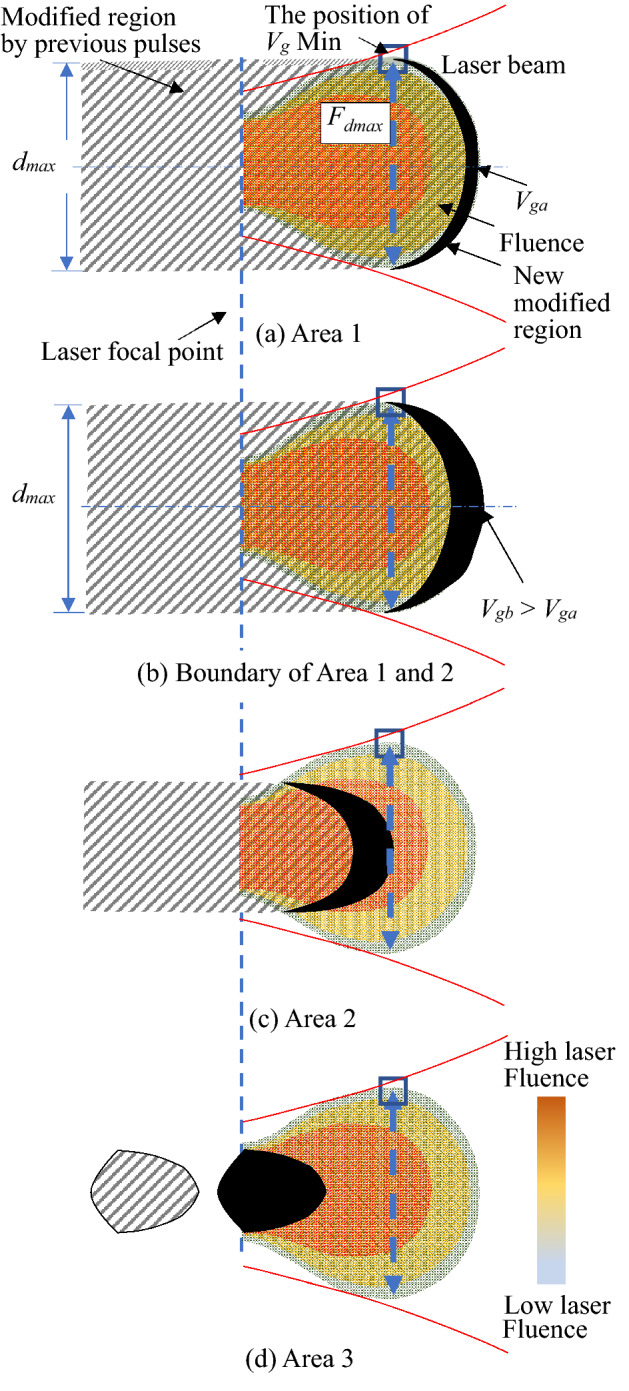
Schematic illustrations of fabrication process of a wire-shaped modified region fabricated at each area shown in Fig. 5.

Kononenko et al. calculated the growth length of the modified region per laser pulse (*V*_*g*_) by observing the modification of each laser pulse in situ. The study indicated that *V*_*g*_ increases with the laser fluence^14^, i.e., *V*_*g*_ depends on the position and has a maximum value at the laser focus, and the minimum at the red line is shown in Fig. 1b when the energy was 2 µJ. In this study, the value of *V*_*f*_ was used as the experimental parameter for the wire-shaped modified region. The fabricated region was connected and almost uniform; therefore, the values of *V*_*f*_ and *V*_*g*_ were in equilibrium in areas 1 and 2. The fabrication point became closer to the laser focus with increasing *V*_*f*_, where the value of *V*_*g*_ increased. Figure 6 presents a schematic of the growth of the wire-shaped modified region in each area. The shapes of the modified regions depended on the fluence distribution of the fabrication threshold, as shown in Fig. 1f. Figure 6 illustrates the relationship between the laser fluence distribution and modified regions. The broken arrow indicates the maximum diameter at which the fluence was higher than the fabrication threshold. As Konnonenko reported that the value of *V*_*g*_ depends on the fabricated point on the laser beam, *V*_*g*_ should be the maximum value on the laser light axis, *V*_*g*_ max. In addition, it should be the minimum value at the edge of the laser, *V*_*g*_ min, as indicated by the blue square in Fig. 6a.

#### Area 1

Figure 6a,b illustrate the modification process in Area 1 and the boundary between Areas 1 and 2, respectively. When *V*_*f*_ was small, the laser focus was located inside the modified region fabricated by the previous pulse. Laser light was absorbed by the tip of the modified region, that is, the defocus position, because the modified regions are black and absorb light efficiently. In the case of Fig. 6a, fabrication also occurs at *F*_*dmax*_ constantly because of the minimum of *V*_*g*_ > *V*_*f*_. In Fig. 6b, the minimum of *V*_*g*_ = *V*_*f*_ and modification also occurs at *F*_*dmax*_. Here, *V*_*ga*_ and *V*_*gb*_ are defined as *V*_*g*_ in Fig. 6a,b, respectively. The relationship between these values is *V*_*gb*_ > *V*_*ga*_; hence, the modified region in Fig. 6b was fabricated closer to the laser focus, that is, a higher laser fluence than that in Fig. 6a. The electrical conductivity in Fig. 6b is higher than that in Fig. 6a because of the higher laser fluence. In conclusion, the diameter of the modified wire-shaped regions is constant at the maximum value, regardless of the value of *V*_*f*_. In contrast, the electrical conductivity increased with the focus movement distance per pulse of *V*_*f*_ because the modified region with a larger *V*_*f*_ was fabricated under a higher laser fluence.

#### Area 2

The modified region was not fabricated at *F*_*dmax*_ in area 2, as shown in Fig. 6c, because of the following conditions: *V*_*g*_ max > *V*_*f*_ > *V*_*g*_ min. The fabricated point was closer to the laser focus, where the value of *V*_*g*_ increased with *V*_*f*_. The diameter of the modified wire-shaped region decreased depending on the fluence distribution. The electrical conductivity of the wire-shaped modified region increases with a larger *V*_*f*_ because the modified region was fabricated using a high laser fluence, as shown in Fig. 6a,b.

#### Area 3

The modification process in this area is illustrated in Fig. 6d under the condition of *V*_*f*_ > *V*_*g*_ max. The laser focus did not overlap with the modification formed by the previous pulse and the modified region did not absorb the pulse. The dotted modified region was fabricated intermittently, as shown in Fig. 4e. The diameter of the dotted modified region became constant at a minimum value, regardless of the value of *V*_*f*_, because it depends on the shape of the fluence distribution of the fabrication threshold at the laser focus. The conductivity decreased significantly because the modification was not connected.

A detailed study on why the modified wire-shaped regions have different conductivities is planned for future work.

## Conclusion

Internally modified regions fabricated via focused picosecond laser illumination inside single-crystal diamonds were studied. The relationship between the diameter/electrical conductivity of the modified regions and the laser fluence distribution was investigated. *V*_*f*_ was used to plot conductivity. As a result, the conductivity increases with *V*_*f*_, suggesting that a higher laser fluence (higher value of *V*_*f*_) results in more sp^2^ bonds from sp^3^ bonded diamond. The detailed results are as follows:

- The diameter and length of the short modified regions fabricated using the ps laser without scanning were matched to the laser fluence-distribution calculation.
- The effect of heat accumulation was investigated by fabricating modified wire-shaped regions with a constant *V*_*f*_ by changing the laser repetition rate and scanning speed. The repetition rate did not affect the shape and conductivity of the wire-shaped modified region at repetition rates of 10–400 kHz.
- The shape and conductivity of the wire-shaped modified regions with various *V*_*f*_ values were studied. The electrical conductivity of the wire-shaped modified region increased with *V*_*f*_ regardless of the constant diameter of the wire-shaped modified region under *V*_*f*_ = 0.16 μm/pulse. The diameter decreases as the value of *V*_*f*_ increases. When *V*_*f*_ was larger than 4.84 μm/pulse, the diameter of the wire-shaped modified region became constant at a minimum value because of the intermittently fabricated dotted-modified region, where the electrical conductivity also decreased significantly.

## Methods

**Figure 7:**
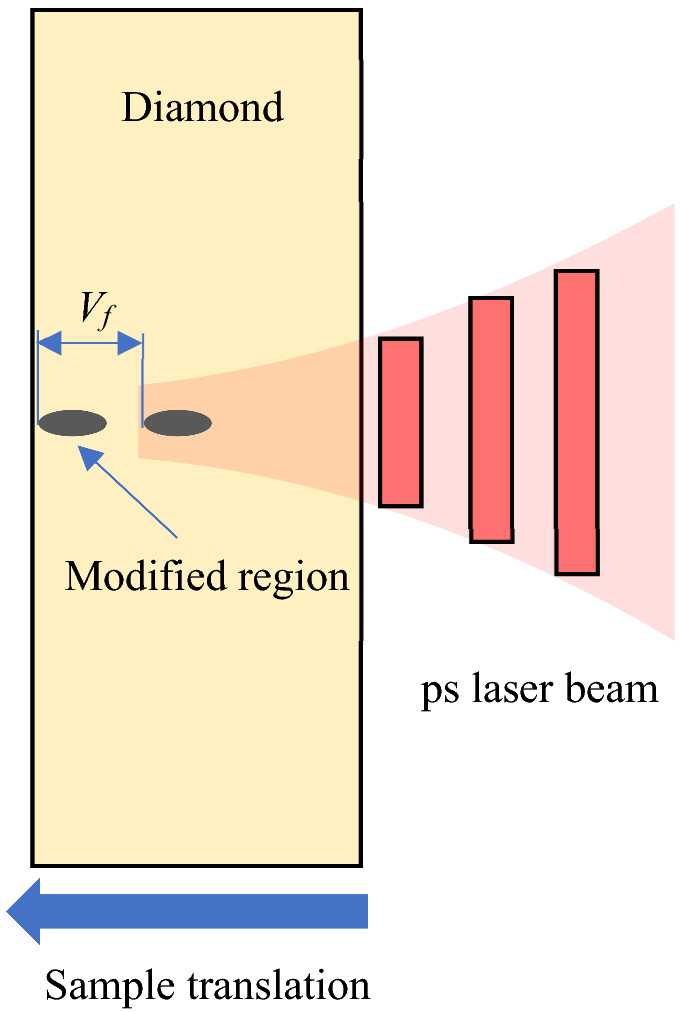
Schematic illustration of experimental apparatus.

The diamond sample was internally modified using a ps laser with the wavelength of 1064 nm, pulse width of 11.3 ps, maximum power of 25 μJ, repetition rate of 10–400 kHz, and a Gaussian-like spatial form with M^2^ < 1.5^20^. The laser beam was focused using an objective lens (M-PLAN NIR 20×; Mitutoyo, Kanagawa, Japan) with the NA of 0.4. Figure 7 displays a schematic of the internal processing of diamond. A high-pressure, high-temperature diamond sample (SUMICRYSTAL UP282512, 3 mm × 3 mm × 1 mm, consisting of (100) planes; Sumitomo Electric Industries, Ltd., Osaka, Japan) was fixed with double-sided tape on a five-axis stage. All the surfaces of the samples were mirror-polished. A focused laser spot was scanned inside the diamond by the stage. The value of *V*_*f*_ shown in Fig. 7 was defined as the movement distance of the focused laser spot per laser pulse inside the diamond; the stage scanning speed (μm/s) was divided by the laser repetition rate (Hz) and multiplied by 2.42 to account for the diamond refraction index.

This study conducted two types of experiments: irradiation with and without laser-focus scanning. In the experiment without scanning, the laser focus was 700 μm on the front surface of the sample. The sample was illuminated by a laser until the growth of the modified region stopped. The laser repetition rate was set to 100 kHz and the laser pulse energy was changed to 0.5–2.0 μJ/pulse. The length and diameter of these modified regions were compared with the cross section along the optical axis of the calculated laser fluence distribution.

**Figure 8:**
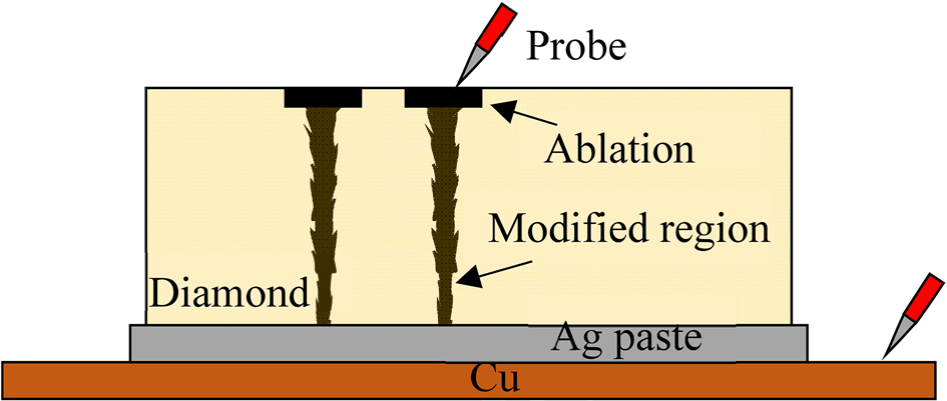
Schematic illustration of a sample conductivity measurement.

In the scanning experiment, the focus was scanned along the laser beam axis from the rear surface to the front surface of the sample to fabricate a modified wire-shaped region. The laser pulse energy was set at 2.0 μJ. The value of *V*_*f*_ was set by changing the focal scanning speed and laser repetition rate. Figure 8 shows the method used to measure the resistance of the modified wire-shaped region. The front surface near the modified region was graphitized by nanosecond laser pulses because it was difficult to adjust the location of the probe tip to contact an exposed area of dozens of micrometers. The modified region was connected to the ablated area and used to measure the electrical conductivity of the ablated area. The resistivity of the ablated area was ~ 200 Ω. The value was sufficiently small to be ignored compared with the modified region, which was on the order of thousands of ohms. The rear surface was connected to a copper plate using Ag paste (Dotite, XA-436, Fujikura Kasei Co., Ltd., Tochigi, Japan) and sintered by atmospheric heating at 150 °C for 30 min. The electrical conductivity between the ablated area and copper plate was measured using a multimeter.

## Data Availability

All relevant data are available upon request from H.H.
